# Repeatability of ^18^F-FDG uptake in metastatic bone lesions of breast cancer patients and implications for accrual to clinical trials

**DOI:** 10.1186/s13550-024-01093-7

**Published:** 2024-03-27

**Authors:** Mark Muzi, Lanell M. Peterson, Jennifer M. Specht, Daniel S. Hippe, Alena Novakova-Jiresova, Jean H. Lee, Brenda F. Kurland, David A. Mankoff, Nancy Obuchowski, Hannah M. Linden, Paul E. Kinahan

**Affiliations:** 1 Department of Radiology, University of Washington Medical Center, 1959 NE Pacific Street, UW Box 356465, Seattle, Washington, 98195 USA; 2 Thomayer University Hospital, Prague, Czech Republic; 3 University of Pennsylvania, Philadelphia, PA USA; 4 Cleveland Clinic, Cleveland, OH USA

**Keywords:** Breast cancer, Bone metastases, ^18^F-FDG PET, Test-retest, Repeatability

## Abstract

**Background:**

Standard measures of response such as Response Evaluation Criteria in Solid Tumors are ineffective for bone lesions, often making breast cancer patients that have bone-dominant metastases ineligible for clinical trials with potentially helpful therapies. In this study we prospectively evaluated the test-retest uptake variability of 2-deoxy-2-[18F]fluoro-D-glucose (^18^F-FDG) in a cohort of breast cancer patients with bone-dominant metastases to determine response criteria. The thresholds for 95% specificity of change versus no-change were then applied to a second cohort of breast cancer patients with bone-dominant metastases.

**Methods:**

For this study, nine patients with 38 bone lesions were imaged with ^18^F-FDG in the same calibrated scanner twice within 14 days. Tumor uptake was quantified by the most commonly used PET parameter, the maximum tumor voxel normalized by dose and body weight (SUVmax) and also by the mean of a 1-cc maximal uptake volume normalized by dose and lean-body-mass (SULpeak). The asymmetric repeatability coefficients with confidence intervals for SUVmax and SULpeak were used to determine the limits of ^18^F-FDG uptake variability. A second cohort of 28 breast cancer patients with bone-dominant metastases that had 146 metastatic bone lesions was imaged with ^18^F-FDG before and after standard-of-care therapy for response assessment.

**Results:**

The mean relative difference of SUVmax and SULpeak in 38 bone tumors of the first cohort were 4.3% and 6.7%. The upper and lower asymmetric limits of the repeatability coefficient were 19.4% and − 16.3% for SUVmax, and 21.2% and − 17.5% for SULpeak. ^18^F-FDG repeatability coefficient confidence intervals resulted in the following patient stratification using SULpeak for the second patient cohort: 11-progressive disease, 5-stable disease, 7-partial response, and 1-complete response with three inevaluable patients. The asymmetric repeatability coefficients response criteria for SULpeak changed the status of 3 patients compared to the standard Positron Emission Tomography Response Criteria in Solid Tumors of ± 30% SULpeak.

**Conclusion:**

In evaluating bone tumor response for breast cancer patients with bone-dominant metastases using ^18^F-FDG SUVmax, the repeatability coefficients from test-retest studies show that reductions of more than 17% and increases of more than 20% are unlikely to be due to measurement variability. Serial ^18^F-FDG imaging in clinical trials investigating bone lesions in these patients, such as the ECOG-ACRIN EA1183 trial, benefit from confidence limits that allow interpretation of response.

**Supplementary Information:**

The online version contains supplementary material available at 10.1186/s13550-024-01093-7.

## Introduction

Breast cancer is the most common malignancy and second leading cause of cancer death in women [1], and bone is the most common site of metastasis in breast cancer [2–5]. The appearance and behavior of bone metastases can be detected on a wide variety of clinical imaging studies (e.g. x-ray computed tomography, bone scan, magnetic resonance imaging, (2-deoxy-2-^18^F-fluoro-D-glucose) ^18^F-FDG using positron emission tomography with computed tomography attenuation mapping (PET/CT [6]). that are performed for different indications.

Imaging-based response criteria are often used to determine the efficacy of new therapeutic agents in cancer treatment trials. The most commonly used set of criteria in clinical trials is the Response Evaluation Criteria in Solid Tumors version 1.1 (RECIST) [7], which focuses predominantly on the physical dimensions of solid tumors from CT scans, similar to other size-based criteria such as those from the World Health Organization (WHO) [8]. However, CT does not evaluate the bone or bone marrow, but only the osteoblastic reaction in healing bone [9]. For this reason, RECIST criteria specify that bone lesions without soft-tissue components are non-measurable, non-target lesions. As a result, patients with bone dominant disease are often excluded from clinical trials due to a lack of RECIST measurable disease [10–12].

There is active interest in using measures of ^18^F-FDG uptake with PET/CT imaging as a biomarker to assess early response to therapy for multiple types of cancer [13, 14]. For breast cancer, the AVATAXHER trial [15] and recently, the 2019 results of the TBCRC026 trial along with at least 11 other studies [6, 16–26], support using PET imaging as an effective method of measuring early breast cancer response in vivo.

An early effort to define PET-based response criteria for clinical trials was led by the European Organization for Research and Treatment of Cancer (EORTC) in 1999 [27]. The EORTC response criteria were expanded and modified by Wahl and colleagues in 2009 for the Positron Emission Tomography Response Criteria in Solid Tumors, or PERCIST [28]. Multiple clinical studies have shown that response assessment by EORTC criteria and PERCIST leads to similar response classifications [29]. In addition, there are preliminary data that suggest that response assessment by PERCIST is better correlated with patient outcome and may be a better predictor for the effectiveness of new anti-cancer therapies than RECIST [30]. However there have only been very limited reported evaluations of the use of PET imaging specifically for response assessment of osseous metastases from breast cancer [9, 31], and an extension of PERCIST to metastatic bone disease is not yet established [21]. Peterson et al. [32] evaluated a modified version of PERCIST inclusion criteria (mPERCIST) accounting for the lower standardized-uptake-values (SUVs) of osseous lesions compared to the soft-tissue lesions previously studied using PERCIST. This study found that changes in ^18^F-FDG-PET uptake during therapy were predictive of time to skeletal-related events (tSRE) and time-to-progression (TTP).

To design effective response criteria, an understanding of the test-retest variability is needed. In this study we prospectively evaluated the test-retest variability of ^18^F-FDG-PET uptake in a cohort of breast cancer patients with metastatic bone-dominant lesions (BD-MBC) using the mPERCIST inclusion criteria. The calculated thresholds for 95% specificity of change versus no-change from the test-retest data were then applied to a second cohort of BD-MBC patients who had ^18^F-FDG-PET scans both pre-therapy and after start of therapy. The classifications of change status were compared to those using EORTC, PERCIST, and recently published thresholds for soft-tissue cancers from the QIBA Profile [33].

## Materials and methods

### Patient selection

#### Cohort-1

Repeatability was assessed in a cohort of nine stage IV BD-MBC patients with stable bone disease that underwent two ^18^F-FDG PET/CT studies on the same scanner within a two-week duration or less with no interval change in therapy. Patient and scan characteristics for cohort-1 appear in Table 1.

**Table 1:** Cohort-1 test-retest patient characteristics

		Bone	Breast			^18^F-FDG Scan Differences^§^			
		Lesions	Cancer	ER/PR/Her2^†^	Treatment	∆Days	∆UT	∆%Dose	∆[Glc]
Case	Age	(n)	Type*	Status	Therapy^‡^	(days)	(min)	(%)	(mg/dL)
01	51	9	IDC	+/+/+	Horm/HDT	14	3.0	-13%	5.0
02	55	2	IDC	+/+/=	Chemo	8	1.0	0%	-14.3
03	60	9	IDC	-/-/+	Chemo/HDT	8	0.0	-7%	1.0
05	38	1	IDC	+/+/=	Not Started	2	0.0	2%	-14.0
06	62	7	Mixed	+/+/-	Chemo	7	0.0	3%	-3.7
07	52	3	IDC	+/+/-	Horm	14	0.0	-3%	-6.7
18	67	3	DCIS	+/-/-	Not Started	12	6.0	-6%	19.0
24	32	4	Unk	+/-/+	Horm/HDT	8	3.0	1%	7.0
26	57	1	IDC	+/+/-	Horm	7	-4.0	-16%	4.0
Mean	51	5	-	-	-	9	1.0	-4%	-0.3
SD	11	3	-	-	-	4	2.8	7%	10.7

^*^Type of cancer is invasive ductal carcinoma (IDC), mixed lobular and ductal carcinoma (Mixed), ductal carcinoma in situ (DCIS) or unknown (Unk); ^†^ER/PR/Her2 pathology status is positive (+), negative (-) or equivocal (=); ^‡^Treatment is endocrine-based or hormonal (Horm), chemotherapeutic (Chemo) or HER2-directed therapy (HDT). ^§18^F-FDG scan differences are ∆Days is the days between scans, ∆UT is the difference in minutes of uptake times (time between injection and scanning) between scans, ∆%Dose is the percent difference in dose between scans and ∆[Glc] is the change in blood glucose concentration between scans

#### Cohort-2

A second retrospective cohort of 28 BD-MBC patients with planned standard-of-care therapy (including endocrine therapy, chemotherapy, and biological therapies) were imaged with ^18^F-FDG before and within 30 days following therapy. Aspects of this study have been presented elsewhere [32].

#### Ethics and Consent

Patients in both cohorts were recruited from the Seattle Cancer Care Alliance or the University of Washington Medical center (Seattle, WA), and signed informed consent prior to enrollment. All methods were performed in accordance with the ethical standards as laid down in the Declaration of Helsinki and its later amendments or comparable ethical standards, as approved by our local IRB (Institutional Review Board), Human Subjects and Radiation Safety committees.

#### PET/CT scanners and calibration

There were three PET scanners used in the study. Cohort-1 patients were all imaged on one of two General Electric (GE) Discovery STE PET/CT scanners [34], with identical reconstruction parameters, where each test-retest study was acquired on the same scanner. In addition to the recommended PET scanner calibration [33], the two scanners were cross-calibrated and quantitative performance was monitored with NIST-traceable reference sources to ensure similar quantitative accuracy [35, 36].

Most cohort-2 patients (15) were imaged on the same PET/CT scanner in serial studies. However, due to the addition of the GE Discovery STE PET/CT scanners at our center, thirteen cohort-2 patients were initially imaged on a GE Advance PET scanner [37] and underwent the second scan on a Discovery scanner. We have shown that our calibration and cross-calibration procedures and identical acquisition and reconstruction protocols provide test–retest accuracy comparable to a well-calibrated single scanner [38].

#### ^18^F-FDG-PET imaging protocol

The imaging protocol was performed according to clinical standards, consistent with the QIBA ^18^F-FDG-PET/CT Profile [33]. Patients fasted for a minimum of 6 h before administration of ^18^F-FDG. Medications that affect bone marrow uptake of the tracer (G-CSF, Epogen, or Procrit) were withheld for 2–3 weeks prior to scanning. The ^18^F-FDG dose, obtained from Cardinal Health, ranged from 260 to 407 MBq (median 350MBq). Images were acquired with a target of 60 min after injection of ^18^F-FDG (actual range 50–70 min) using multiple fields-of-view to image from the level of the eye orbits to mid-thigh.

#### Image analysis

Images from the Advance PET scanner were reconstructed using 2D filtered back projection reconstruction (4.29 × 4.29 × 4.25 mm voxel resolution), while images from the Discovery PET/CT scanners used iterative 3D reconstruction (4.29 × 4.29 × 3.27 mm voxel resolution). All reconstructions had corrections for dead time, random events, scatter, sensitivity, decay, branching ration, and attenuation. PET images were read by two qualified and experienced nuclear medicine physicians.

Quantitative uptake values (kBq/cc) for each lesion were extracted using the PMOD image analysis software (PMOD Technologies V4.1, Zurich, CH). SUVpeak volumes-of-interest (VOIs) were constructed as a cubic volume of approximately 1.5 cc centered on the maximum voxel (SUVmax) of each bone lesion. The average SUV of the VOI was the SUVpeak value. Both SUVmax and SUVpeak were normalized to lean-body-mass producing SULmax and SULpeak.

#### Statistical methods

Repeatability of SUV/SULs in metastatic bone lesions in cohort-1 patients was assessed using the procedures described by Velasquez et al. for gastrointestinal cancers [39] and Weber et al. for non–small cell lung cancer [40]. Both studies used ^18^F-FDG-PET multicenter test-retest exams, as in the current study. A description of the calculated metrics is summarized in Supplementary materials Table S1. Variability was assessed by calculating the difference of paired measurements, and the difference of the logs of the measurements using SUV to reflect SUVmax or SULpeak:

$${d}_{i}={\text{S}\text{U}\text{V}}_{i,2}-{\text{S}\text{U}\text{V}}_{i,1}$$

$${\varDelta }_{i}=ln\left({\text{S}\text{U}\text{V}}_{i,2}\right)-ln\left({\text{S}\text{U}\text{V}}_{i,1}\right)=ln\left(\frac{{\text{S}\text{U}\text{V}}_{i,2}}{{\text{S}\text{U}\text{V}}_{i,1}}\right)$$

The difference of the log of the measurements, *∆*_*i*_, can be useful where *d*_*i*_ does not follow a normal distribution or where the relative differences are found to be proportional to the mean [41]. The SUV measurements SUV_*i*1_ and SUV_*i*2_ are for lesion *i* at the time of the baseline and the follow-up scans, and are calculated using SUVmax and SULpeak, which are the most common clinical ^18^F-FDG-PET biomarkers. The variability of the parameters *d*_*i*_ and the log-transformed values, *∆*_*i*_, were assessed using Bland-Altman plots. The consistency of *d*_*i*_ and *∆*_*i*_ with a normal distribution were assessed with quantile–quantile plots and Kolmogorov–Smirnov tests.

The log-transformed data were used to calculate the mean percent difference in uptake between scans (%$\stackrel{-}{{\Delta }}$), within-subject coefficient of variation ($w\text{C}{\text{V}}_{{\Delta }}$), the repeatability coefficient (RC), and asymmetric RC limits (-RC and + RC) as described in Supplementary materials, Table S1. The 95% confidence interval (CI) for %$\stackrel{-}{{\Delta }}$ (an estimate of bias between scans) did not include 0. However, this was hypothesized to be a sampling effect and to be conservative, the repeatability metrics were also calculated without subtracting the sample mean. This will include any bias into the estimate of variability and increases the associated metrics: the within-subject coefficient of variation with bias included ($w\text{C}{\text{V}}_{{\Delta }0}$), the repeatability coefficient with bias included (RC_0_) and the asymmetric repeatability coefficients with bias included (-RC_0_ and + RC_0_). Details of the calculations are provided in the Supplementary materials.

Metrics were calculated using the lesion as the unit of analysis. To account for non-independence of multiple lesions from the same patient, 95% CIs for the repeatability coefficients were calculated using the leave-one-patient-out jackknife method [42]. This involves estimating the standard error of the repeatability metric by recalculating the metric after one patient at a time (all lesions from that are excluded at each step) as this assumes the patients are independent but the lesions within patient are not. The Supplementary materials describes the approach in more detail.

#### PERCIST Quality Control

We applied the PERCIST recommendations for quality control by measuring the mean SUL of a 3 cm spherical VOI in a normal region of the right lobe of the liver to check that the difference between the scans is less than 20% and less than a SULmean value of 0.3 for both cohort-1 and cohort-2 patients.

#### Inclusion criteria

The PERCIST criteria for including lesions in evaluations of response to therapy is $\text{S}\text{U}\text{L}\text{p}\text{e}\text{a}\text{k} \ge 1.5\bullet {r}_{L}+2\bullet {s}_{L}$, where ${r}_{L}$ is the mean SUL value of the normal liver region described above and ${s}_{L}$ is the sample standard deviation of the VOI. As we have previously noted [32, 43], bone lesions appear to have lower average SULpeak values and lower coefficient of variation than soft-tissue lesions previously studied using PERCIST. In addition, it has been shown that the standard deviation of a VOI from a single image is not related to the true noise, i.e. the noise measured from multiple images of the same object [44]. For these reasons we proposed a modified PERCIST (mPERCIST) lesion inclusion criteria for bone lesions defined by liver $\text{S}\text{U}\text{L}\text{p}\text{e}\text{a}\text{k} \ge 1.5\bullet {r}_{L}$.

Cohort-2 patient data was used to assess the impact of PERCIST and mPERCIST thresholds for inclusion in studies, as well as the use of cohort-1 bone lesion ± RC for the determination of response to therapy. The PERCIST approach uses the concept of a ‘target’ lesion to determine response, where only the percentage difference in SULpeak between the tumor with the highest value in study 1 and the tumor with the highest value in study 2 (i.e. not necessarily the same tumor) is used as the classifier for response. The criteria from EORTC and QIBA were also included where appropriate.

## Results

### Cohort-1 characteristics

Nine female breast cancer patients were enrolled in cohort-1 with an average age of 51 years (median 55, range 32–62) with metastatic bone disease. Patients had a mixture of sclerotic, lytic, or mixed-type lesions. Most of the patients were postmenopausal (7/9, 78%) with invasive ductal carcinoma (6/9, 67%). Most patients had ER positive disease (8/9, 89%), while some were HER2 negative (4/9, 44%). Seven patients were on therapy before enrolling in the study, and two had no therapy prior to the repeatability scans. For the patients that were on treatment, there were no changes to treatment between the two scans. The injected dose of ^18^F-FDG ranged from 305 to 396 MBq for both test and retest scans (mean 368 MBq ± 20 MBq). The median time between scans was 8 days (range 2–14). Average glucose level for the first scan was 94 mg/dL (range 88–104) and for the second scan was 92 mg/dL (range 89–96). The uptake time from tracer injection to the onset of imaging averaged 61 min (range 58–70 min for all scans), while the difference in uptake times between scan1 and scan2 per patient ranged from 0 to 6 min. Cohort-1 patient and scan characteristics appear in Table 1.

### Repeatability of bone lesion ^18^F-FDG uptake values

**Fig. 1:**
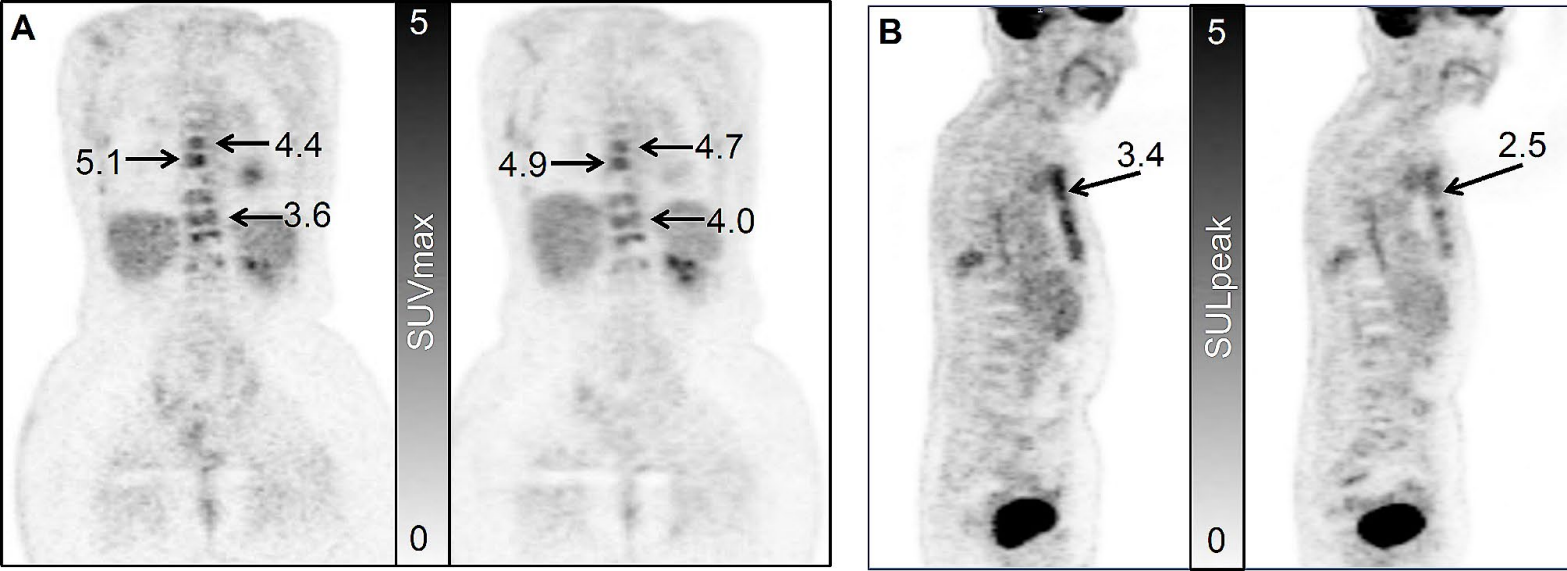
(**A**) Cohort-1 study. Coronal ^18^F-FDG-PET images from the same patient imaged 7 days apart with SUVmax values indicated. (**B**) Cohort-2 study. Sagittal ^18^F-FDG-PET images of a 90-year old female with bone-dominant MBC. Left: Pre-therapy baseline scan showing the SULpeak of the index lesion. Right: Post therapy 4mo scan, that shows a decrease of 25%, which met our LRC threshold of -18%, but not the PERCIST threshold of -30%. The SUVmax of the index lesion decreased by 22%. The response was considered stable disease by the criteria developed in this report

Individual SUVmax and SULpeak test-retest measurements for 38 lesions from 9 patients in cohort-1 are provided in Table S2 of the Supplementary materials. The median number of lesions per patient was 5 (range: 1 to 9 lesions). An example test-retest ^18^F-FDG image set from cohort-1 is shown in Fig. 1, and illustrates the consistency of SUV measures between the scans. Also shown is a cohort-2 example of response to therapy as assessed by SUV.

**Fig. 2:**
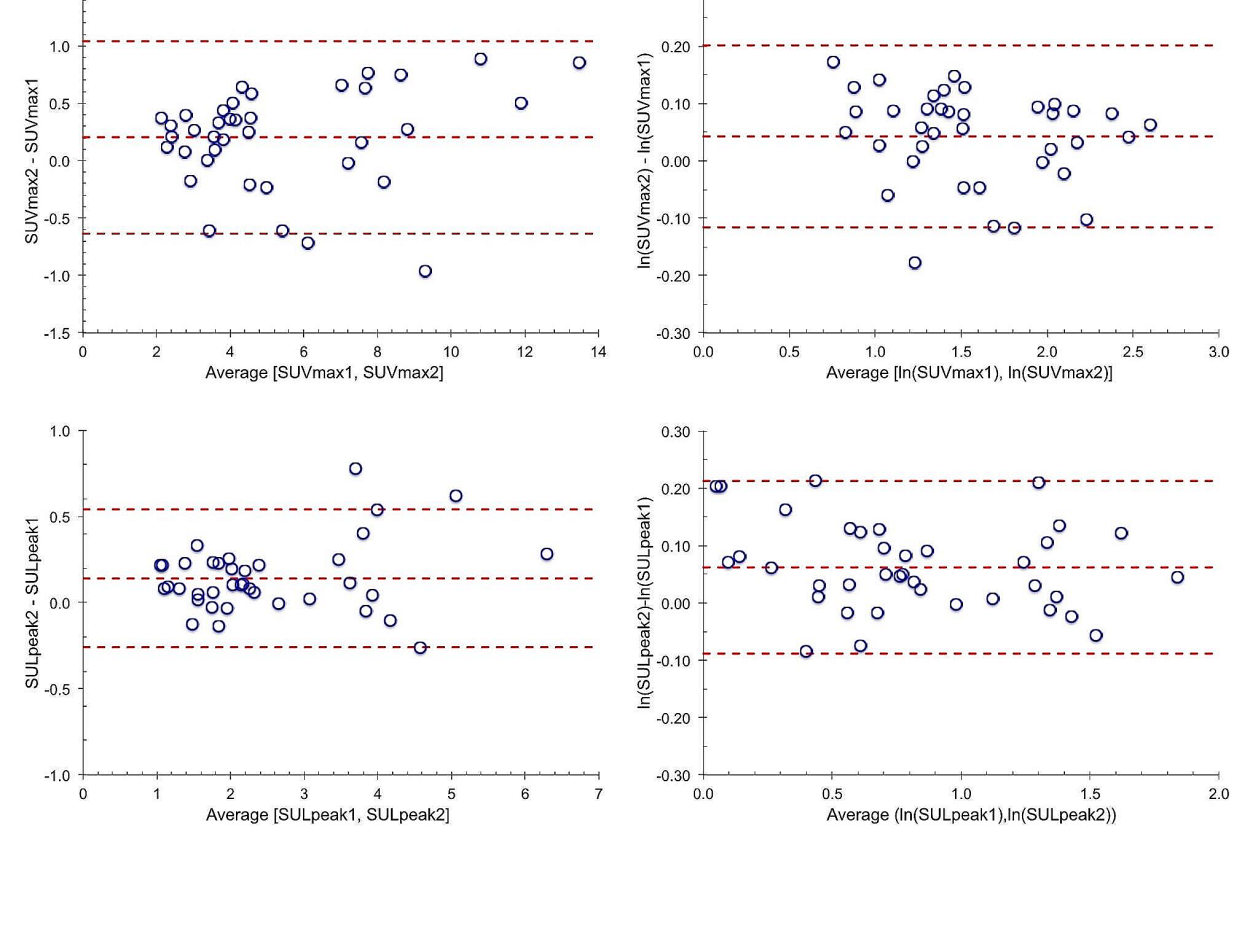
Bland-Altman plots for all 38 lesions for the 9 patients. Top: SUVmax. Bottom: SULpeak. Left: Test-retest difference versus average value. Right: Differences of the natural logarithms. Dashed lines are the mean difference and the upper and lower limits of agreement

For quantitative analysis, Bland-Altman plots of individual lesion differences for SUVmax and SULpeak are shown in Fig. 2. The corresponding Bland-Altman plots for within-patient averages of lesions are shown in Supplementary materials Figure S1.

The tests of normality of the differences, using both quantile-quantile plots (Supplemental materials Figure S2) and Kolmogorov–Smirnov tests (*p* = 0.88), showed that all the results were consistent with a normal distribution. The Bland-Altman plots above indicated a potential increase in variance of the SUV differences as a function of the average value. This dependence was not apparent in the difference of the natural logarithms of the SUV values.

The derived repeatability metrics for metastatic bone lesions in breast cancer patients using log-transformed SUVmax and SULpeak measurements, which are normally distributed, are provided in Table 2. The repeatability metrics for other extracted PET parameters, SUVpeak and SULmax, are presented in Supplementary materials Table S3.

**Table 2:** SUV Repeatability metrics for all *n* = 38 lesions

Parameter	SUVmax	SULpeak
SUVmin, SUVmax	2.0, 13.9	0.9, 6.4
$\stackrel{-}{\varvec{d}}$ ± SD	0.20 ± 0.43	0.14 ± 0.20
*d*_*i*_ Min, Max	-0.96,0.88	-0.26, 0.77
*$\stackrel{-}{\varDelta }$ [-CI, +CI] (%)	4.4 [0.4, 8.3]	6.4 [1.1, 11.7]
-RC [-CI, +CI] (%)	-14.7 [-17.9, -11.3]	-14.0 [-18.6, -9.1]
+RC [-CI, +CI] (%)	17.2 [12.7, 21.8]	16.3 [10.0, 22.9]
${\varvec{w}\mathbf{C}\mathbf{V}}_{\varDelta }$ [-CI, +CI] (%)	5.9 [4.4, 8.3]	5.6 [3.5, 7.7]
-RC_0_ [-CI, +CI] (%)	-16.3 [-19.7, -12.8]	-17.5 [-25.3, -8.8]
+RC_0_ [-CI, +CI] (%)	19.4 [14.6, 24.5]	21.2 [9.7, 33.9]
$\varvec{w}{\mathbf{C}\mathbf{V}}_{\varDelta 0}$ [-CI, +CI] (%)	6.6 [5.0, 8.2]	7.2 [3.4, 11.1]

^*^The 95% confidence interval of the average difference of log-transformed data,${\text{C}\text{I}}_{\stackrel{-}{\varDelta }}$, was found to not contain zero. This was hypothesized to be a sampling effect and RCs were recalculated without subtracting the sample mean, as described in the Supplementary materials. This approach led to a slightly more conservative (i.e. larger) estimates of the repeatability coefficients and coefficient of variation, denoted in Supplementary materials Table S2 as *±* RC_*0*_ and wCV_*∆0*_.

### PERCIST Quality Control: Cohort-1

For cohort-1, the average liver SULmean was 1.6 (range 1.2 to 2.0) in the first scan and 1.6 (range 1.3 to 1.8) in the second scan. The average difference between scans for ^18^F-FDG SULmean in liver was − 0.02 (range − 0.21 to 0.15). The differences in patient liver SUL values between scans were well under the threshold of < 0.3 SULmean suggested by PERCIST guidelines.

### Cohort-2 characteristics

Patient and scanning characteristics of the 28 patients in cohort-2 are presented in Supplemental materials Table S4. After baseline ^18^F-FDG imaging, patients received different therapies before post therapy PET imaging and were followed clinically thereafter. There were 146 metastatic bone tumors identified by a combination of ^18^F-FDG-PET and CT imaging [32].

### PERCIST Quality Control: Cohort-2

For cohort-2, 3 of the 28 patients did not meet the PERCIST quality control requirement (i.e. the difference between scans of the SULmean for a liver ROI was more than 0.3 and greater than 20%), and one patient had uninterpretable liver results from the second scan.

### Assessment of inclusion criteria

**Fig. 3:**
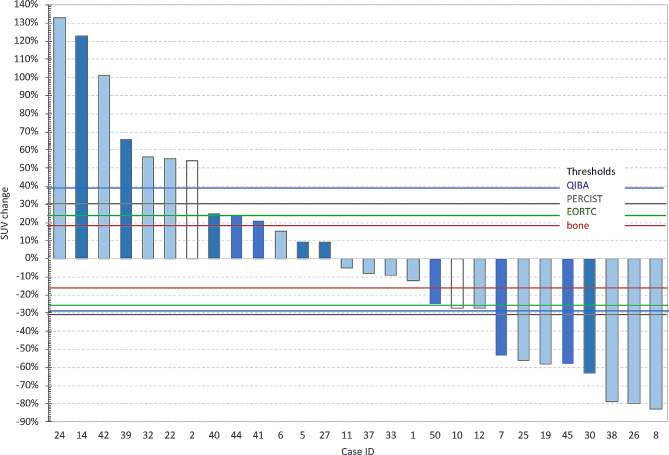
Percentage change in SUVmax for cohort-2 patients ordered by magnitude of change. Dark bars are cases where new lesions appeared in the second PET scan and white bars indicate an issue with initially low SUVmax (1.0-1.8) values. The horizontal lines are the thresholds for classifying a change as determined by the PERCIST, QIBA, EORTC and this study especially for bone metastases

The PERCIST threshold allowed assessment of 23 patients, and the mPERCIST threshold allowed assessment of 26 patients of the 28 patients in the cohort (Supplementary materials Table S5). We note that of the 3 additional patients included by the mPERCIST criteria, one did not meet the PERCIST quality control requirement for liver (Case 50 in Table S5). While the PERCIST approach uses only the change in the single target lesion(s) to determine response, we also evaluated the impact of the change in inclusion thresholds on all 146 metastatic lesions in the 28 patients and found that the PERCIST threshold allowed assessment of 76 of the bone tumors (52%). The mPERCIST threshold allowed assessment of 102 (70%) of the bone tumors, a substantial increase. These changes for the target lesion ^18^F-FDG SUVmax are illustrated in Fig. 3 along with thresholds for partial response (PR) and progressive disease (PD) based on QIBA, PERCIST, EORTC and the ± RC_0_ threshold developed from cohort-1 test-retest study (-RC_0_ = -16.3% for PR and + RC_0_ = 19.4% for PD). The ± RC values using SULpeak are -RC_0_ = -17.5% for PR and + RC_0_ = 21.5% indicating PD.

### Assessment of Response thresholds

The response criteria developed from cohort-1 test-retest studies of ^18^F-FDG SULpeak values in bone lesions (± RC_0_) changed the response status of 4/28 patients compared to standard PERCIST response criteria. The changes were evenly divided between shifts from stable disease (SD) to progressive disease (PD) or to partial response (PR) when shifting from the PERCIST thresholds of ± 30% to the bone metastasis ± RC threshold of change (-17.5%, + 21.2%) for ^18^F-FDG SULpeak values. In some cases new lesions appear, which is considered an overriding determination of progressive disease, regardless of the change in SUL, PERCIST/mPERCIST threshold or PERCIST inclusion criteria.

## Discussion

Our primary finding, albeit based on a study of 9 patients with a total of 38 metastatic bone lesions, was that the test-retest variability of ^18^F-FDG uptake in bone is lower than has been previously published for soft-tissue tumors [39, 40, 45–47] or mixed tumors typical of breast cancer recurrence [36]. As summarized in the QIBA Profile summary paper [33], the within-subject coefficient of variation ranged from 10 to 12% in the above cited publications. In our study we estimated a within-subject coefficient of variation (wCV_*∆*_) for SUVmax of 6.6% (95% CI: 5.0–8.2%) and for SULpeak of 7.2% (95% CI: 3.4–11.1%). There are two implications from this reduction in variability: First that inclusion criteria can be relaxed compared to the EORTC, PERCIST, and QIBA proposals. Second, that the thresholds for determining response can also be reduced. These comparisons are described in Table 3.

**Table 3:** , Comparison of EORTC and PERCIST response criteria, QIBA Profile Claims and current study

Category	EORTC criteria	PERCIST criteria	QIBA Profile Claim	This study (mPERCIST)
Measurable lesions	Region of high ^18^F-FDG uptake representing viable tumor	SULpeak (hottest tumor) ≥ (1.5 x mean liver uptake + 2 SD of liver region)	SUVmax ≥ 4 and diameter ≥ 2 cm	SULpeak (hottest tumor) ≥ (1.5 x mean liver uptake)
No change condition (stable disease for EORTC and PERCIST)	Increase in SUV < 25% or decrease of < 15% after one cycle of treatment (and < 25% after more than one treatment cycle) and no change in extent > 20%	Change in SULpeak < ± 30%, If there are other changes that indicate response or progression.	Claim 2: Increase in SUVmax < 39% and decrease in SUVmax < 28%.	SULpeak < 21% or and decrease in SULpeak <-17% or an increase in SUVmax < 19%, and decrease in SUVmax< -16% assuming a mean difference between test and retest of zero.
Conditions for valid measurements	Scanners should provide reproducible data	Normal liver SUL must be within 20% and 0.3 SUL for baseline and follow-up studies	Acquisition and analysis must comply to the QIBA Profile checklists	Same as PERCIST.
Protocol	Specified	Refers to NIH-consensus (40) and Netherlands (41) protocols	Specified	Kurland 2019 and Peterson 2018

Note that this only contains an excerpt of the detailed EORTC and PERCIST response criteria. In addition, the EORTC and PERCIST response criteria are intended to provide information on disease status, while the QIBA Profile Claims are providing information about the statistical variability of SUVs under the assumption of no true biological change. Acronyms. EORTC: European Organization for Research and Treatment of Cancer. PERCIST: PET Response Criteria in Solid Tumors. QIBA: Quantitative Imaging Biomarkers Alliance. ^18^F-FDG: ^18^F-flourodexyglucose. SUV: standardized uptake value. SUVmax,: SUV calculated using the maximum value of a region placed over the image of an ^18^F-FDG-avid lesion. SULpeak,: The mean SUV of a 1 cm diameter region centered over the maximum value of an ^18^F-FDG-avid lesion with biodistribution normalization by lean body mass

As noted above, a small bias in the mean test-retest relative difference was observed for log-transformed SUVmax and SULpeak, where corresponding 95% CIs did not include 0. However, this was thought to be due to sampling variability rather than a true bias between the two scans. To be conservative in the repeatability coefficient estimates, we recalculated the repeatability metrics without subtracting the sample mean, assuming the true bias was zero, which would in effect include the estimated bias as part of the variability and thus somewhat increasing the variability estimates. This increased the estimated within-subject coefficient of variation ($w\text{C}{\text{V}}_{{\Delta }}$) from 5.9 to 6.6%. Justification for assuming a mean relative difference of zero includes; patients were scanned on the identical scanner for test and retest scans and had similar injected doses, blood glucose concentrations and uptake times. Additionally, the soft tissue tumors for these same patients in cohort-1 did not show a bias in test-retest SUV metrics [36], which may be related to the small size but intense ^18^F-FDG uptake in bone metastases.

We did not see a difference in reproducibility for metastatic bone lesion between types of primary breast cancer disease, such as lobular or ductal, however the number of lesions studied was limited and most patients had ductal disease.

## Conclusions

Quantitative ^18^F-FDG-PET SUV uptake values can be highly repeatable measures in breast cancer patients with bone metastases, when acquired in a well-calibrated PET scanner with careful attention to scanner calibration, acquisition protocols and image analysis. This small cohort indicates that repeat bone metastases SUV metrics can be measured with a within-patient COV ($w{\text{C}\text{V}}_{\varDelta 0}$) of less than 8%. In evaluating response assessment in breast cancer patients with bone-dominant metastases, a percentage decrease in ^18^F-FDG SUVmax of more than 17% (SULpeak < 18%) would indicate response, while an increases of more than 20% (SULpeak > 22%) would indicate disease progression, and unlikely to be due to measurement variability. Multicenter clinical trials, such as ECOG-ACRIN EA1183 (FEATURE) trial, assessing metastatic bone disease with ^18^F-FDG PET/CT will directly benefit from (1) a relaxed bone PERCIST threshold for bone tumor assessment, and (2) confidence limits of bone tumor SULpeak or SUVmax that allow interpretation of response to therapy using ^18^F-FDG uptake in bone lesions from breast cancer patients with bone-dominant metastatic disease.

### Electronic supplementary material

Below is the link to the electronic supplementary material.

Supplementary Material 1

## Data Availability

The datasets generated and/or analyzed during the current study are included along with the manuscript submission in the Supporting Matierials section. Raw image data is available upon request.

## Abbreviations

^18^F-FDG: 2-deoxy-2-[^18^F]fluoro-D-glucose
95% CI: 95% Confidence interval
AVATAXHER: A Study of Bevacizumab added to Trastuzumab plus Docetaxel in the Neoadjuvant Setting in Participants With Early Stage HER2-Positive Breast Cancer
BD-MBC: Bone-dominant metastatic breast cancer
ECOG-ACRIN: Eastern Cooperative Oncology Group-American Collage of Radiology Imaging Network
EORTC: European Organization for Research and Treatment of Cancer
ER: Estrogen Receptor
FEATURE ECOG-ACRIN EA1183 clinical trial: FDG PET to Assess Therapeutic Response in Patients with Bone-dominant Metastatic Breast Cancer
G-CSF: Granulocyte colony-stimulating factor
HER2: Human epidermal growth factor receptor 2
IRB: Institutional Review Board
mPERCIST: A modified version of PERCIST inclusion criteria for bone metastases
MBq: Mega-bequerel
mg/dL: Milligrams per deciliter, the clinical units of blood glucose concentration
MRI: Magnetic Resonance Imaging
NIST: National Institute of Standards and Technology
PERCIST: Positron Emission tomography Response Criteria In Solid Tumors
PET/CT: Positron Emission Tomography and Computed Tomography scanning
QIBA: Quantitative Imaging Biomarkers Alliance
RECIST: Response Evaluation Criteria In Solid Tumors
SUV: Standardized Uptake Value normalized by dose and body weight
SUVmax: Maximum voxel in a region in SUV units
SULpeak: Average activity in a ~ 1 cc volume normalized by dose and lean body mass
RC: Repeatability Coefficient
TBCRC026: Phase II Trial Correlating Standardized Uptake Value With Pathologic Complete Response to Pertuzumab and Trastuzumab in breast cancer
tSRE: Time to Skeletal Related Event
TTP: Time To Progression
VOI: Volume of Interest
wCV: Within patient coefficient of variation
WHO: World Health Organization
