# Supplementary material for: Repeatability of 18F-FDG uptake in metastatic bone lesions of breast cancer patients and implications for accrual to clinical trials
Source: EJNMMI Res. 2024 Mar 27;14:32. doi: 10.1186/s13550-024-01093-7 (PMC10973316; doi:10.1186/s13550-024-01093-7)
Supplement: Supplementary file 1 — Supplementary Material 1 [file 13550_2024_1093_MOESM1_ESM.docx]

Repeatability of ^18^F-FDG uptake in metastatic bone lesions of breast cancer patients and implications for accrual to clinical trials

*Mark Muzi, Lanell M. Peterson, Jennifer M. Specht, Daniel S. Hippe, Alena Novakova-Jiresova , Jean H. Lee, Brenda Kurland, David A. Mankoff, Nancy Obuchowski, Hannah M. Linden, Paul E. Kinahan^.^*

Supplementary materials:

**Section 1.** Statistical methods for determining repeatability

Table S1: Term Definitions

| Parameter | Notation | Formulation |
| --- | --- | --- |
| SUVmax | SUVmax | The maximum voxel in a 1.5cc tumor VOI |
| SUVpeak | SUVpeak | The mean activity in a 1.5cc tumor VOI |
| Lesion *i* measurement at scan 1 | SUV*_i_*_1_ | SUVmax or SULpeak |
| Lesion *i* measurement at scan 2 | SUV*_i_*_2_ | SUVmax or SULpeak |
| Number of lesion pairs (SUV*_i_*_1_,SUV*_i_*_2_) | *n* | - |
| Difference of the lesion SUVs of the *i*th pair | *d_i_* | $\mathrm{SUV}_{i2}-\mathrm{SUV}_{i1}$ |
| Mean of the differences for *n* pairs | $\bar{d}$ |  |
| Difference of the log-transformed data pair | *∆_i_* | $ln\left( {\mathrm{SUV}_{i2}}/{\mathrm{SUV}_{i1}} \right)$ |
| Mean difference of log-transformed data | $\bar{\Delta}$ | $\frac{1}{n}\bullet\sum_{i=1}^{n} \Delta_{i}$ |
| Mean percent difference of log-transformed data | $\%\bar{\Delta}$ | $\left( exp\left( \bar{\Delta} \right)-1 \right) \times100\%$ |
| Standard Deviation of ∆*_i_* (the difference of log-transformed data) | *s*_∆_ | $\sqrt{\frac{1}{n-1}\sum_{i=1}^{n} \left( \Delta_{i}-\bar{\Delta} \right)^{2}}$ |
| Standard Deviation of ∆*_i_* (the difference of log-transformed data), with any bias included as variability | *s*_∆0_ | $\sqrt{\frac{1}{n}\sum_{i=1}^{n} \left( \Delta_{i}-0 \right)^{2}}$ |
| Within-subject standard deviation of the log-transformed measurements | _w_*s*_∆_ | ${s_{\Delta}}/\sqrt{2}$ |
| Within-subject standard deviation of the log-transformed measurements with bias included as variability | _w_*S*_∆0_ | ${s_{\Delta0}}/\sqrt{2}$ |
| Within-subject coefficient-of-variation of log transformed values | wCV_∆_ | $\left( exp\left( {}_{w}s_{\Delta} \right)-1 \right) \times100\%$ |
| Within-subject coefficient-of-variation of log-transformed values with bias included as variability | wCV_∆0_ | $\left( exp\left( {}_{w}s_{\Delta0} \right)-1 \right) \times100\%$ |
| Symmetric Repeatability Coefficient | RC_∆_ | $1.96\cdot s_{\Delta}$ |
| Symmetric Repeatability Coefficient with bias included as variability | RC_∆0_ | $1.96\cdot s_{\Delta0}$ |
| Asymmetric limits of RC | ±RC | $\left( exp\left( -{RC}_{\Delta} \right)-1 \right) \times100\%$ |
| Asymmetric limits of RC including bias | ±RC_0_ | $\left( exp\left( +{RC}_{\Delta0} \right)-1 \right) \times100\%$ |
| Limits of agreement on the Bland-Altman plots | LOA | $\bar{\Delta}\pm1.96\cdot s_{\Delta}$ |

**Repeatability metrics calculated including bias as part of the variability.**

Repeatability metrics in this study were defined as in prior ^18^F-FDG PET/CT studies (*1-3)*. As described below, we also calculated variations of these metrics that did not subtract off the sample mean and thus include any estimated bias as part of the variability estimate.

The standard formula for the mean of the differences between the test and retest uptake values, and the standard deviation, *s*_∆_, are:

$\begin{matrix} \bar{\Delta}= & \frac{1}{n}\sum_{i=1}^{n} \Delta_{i} \end{matrix}$

$\begin{matrix} s_{\Delta}= & \sqrt{\frac{1}{n-1}\sum_{i=1}^{n} \left( \Delta_{i}-\bar{\Delta} \right)^{2}} \end{matrix}$

The formula for $s_{\Delta}$ subtracts off the sample mean, so a potential bias (mean of ∆*_i_*) is removed. For example, if any constant value is added to each ∆*i*, it will cancel out in the calculations of $s_{\Delta}$ and have no effect on the result.

The 95% confidence interval of the average difference in log-transformed SUVmax or SULpeak values was found to not contain zero, thus there appears to be a bias, where theoretically there should not have been. So if the true bias is assumed to be zero, any apparent bias (non-zero $\bar{\Delta}$) can be considered part of the variability or deviation from zero. The standard deviation formula can be modified to avoid subtracting the sample mean, $\bar{\Delta}$, where the natural estimator would subtract 0 in place of the sample mean and would be:

$$s_{\Delta0}= \sqrt{\frac{1}{n}\sum_{i=1}^{n} \left( \Delta_{i}- 0 \right)^{2}}=\sqrt{\frac{1}{n}\sum_{i=1}^{n} \left( \Delta_{i} \right)^{2}},$$

where $\bar{\Delta}$ is replaced with 0 and $1/{n-1}$ is changed to $1/n$ since there is no longer a loss of 1 degrees of freedom caused by subtracting off the sample mean.

The repeatability metrics, symmetric and asymmetric repeatability coefficients, based on $s_{\Delta}$ can be then be modified to use $s_{\Delta0}$ instead to reflect the additional variability included, as shown in Table S1. Of note, $s_{\Delta0}$ is nearly always larger than $s_{\Delta}$, so this approach can be considered more conservative in estimating repeatability, resulting in wider RC intervals.

Additionally, the formula for the within-subject standard deviation that includes bias as a part of the variability, ${}_{w}s_{\Delta0}$, can also be calculated by using the formula:

${}_{w}s_{\Delta0}= {s_{\Delta0}}/\sqrt{2}$

The within subject coefficient of variation, ${}_{w}\mathrm{CV}_{d}$, can then be expressed as a percentage:

$${}_{w}\mathrm{CV}_{d}=\left( exp\left( {}_{w}s_{\Delta} \right)-1 \right) \times100\%$$

**Leave-one-patient-out jackknife to calculate 95% confidence intervals**

Repeatability metrics were calculated using the lesion as the unit of analysis. However, the 9 patients in cohort-1 contributed 38 lesions in total (median: 3 lesions / patient; range: 1-9 lesions/patient). The lesions from the same patient are not necessarily independent.

To calculate 95% confidence intervals (CIs), the non-independence of multiple lesions from the same patient need to be accounted for. Therefore, we used the leave-one-patient-out jackknife to estimate the standard error (SE) and compute 95% CIs that are valid for this type of non-independent (clustered) data (*4)*. This involves recalculating the repeatability metric after leaving each patient out one at a time (excluding all lesions from that patient) and combining the results. Specifically, the standard error formula for the jackknife is:

$$SE_{m}= \sqrt{\frac{n-1}{n}\sum_{i=1}^{n} \left( m_{i}-\bar{m} \right)^{2}}$$

where $SE_{m}$ is the standard error of the repeatability metric $m$, n = number of patients, $m_{i}$ is the value of the repeatability metric after excluding patient *i*, and $\bar{m}=1/n\sum_{i=1}^{n} m_{i}$. The 95% CI was then calculated as

$$CI_{m}=m_{i}\pm q_{t, 0.95,n-1}\times SE_{m}$$

where $q_{t,0.95,n-1}$ is the upper quartile of the t-distribution with n-1 degrees of freedom to achieve a 95% two-sided CI (i.e., the 97.5^th^ percentile). In this study with n = 9 patients in cohort-1, $q_{t,0.95,8}\cong2.3$. This quartile was used to help compensate for the fact that $SE_{m}$ was estimated using only 9 independent units (patients), with the loss of 1 degree-of-freedom due to also estimating the sample mean.

**Section 2:** Patient information and image data measurements

Table S2: Cohort-1 SUVmax and SULpeak test-retest measurements for 38 lesions from 9 patients.

| Lesion | Patient ID | SUVmax1 (g/ml) | SUVmax2 (g/ml) | SULpeak1 (g/ml) | SULpeak2 (g/ml) |
| --- | --- | --- | --- | --- | --- |
| 1 | 1 | 13.07 | 13.92 | 6.16 | 6.44 |
| 2 | 1 | 10.39 | 11.27 | 4.76 | 5.38 |
| 3 | 1 | 9.79 | 8.82 | 4.72 | 4.45 |
| 4 | 1 | 8.70 | 8.97 | 4.22 | 4.12 |
| 5 | 1 | 8.27 | 8.08 | 3.91 | 3.95 |
| 6 | 1 | 7.50 | 7.65 | 3.61 | 4.00 |
| 7 | 1 | 7.38 | 8.14 | 3.31 | 4.09 |
| 8 | 1 | 7.23 | 7.21 | 3.57 | 3.68 |
| 9 | 1 | 6.70 | 7.36 | 3.35 | 3.60 |
| 10 | 2 | 4.30 | 4.88 | 2.22 | 2.30 |
| 11 | 2 | 7.36 | 7.99 | 3.87 | 3.82 |
| 12 | 3 | 5.13 | 4.89 | 2.67 | 2.66 |
| 13 | 3 | 4.40 | 4.65 | 2.12 | 2.23 |
| 14 | 3 | 4.39 | 4.75 | 2.28 | 2.50 |
| 15 | 3 | 4.01 | 4.65 | 2.10 | 2.19 |
| 16 | 3 | 3.82 | 4.18 | 1.98 | 2.08 |
| 17 | 3 | 3.75 | 3.14 | 1.56 | 1.43 |
| 18 | 3 | 3.73 | 3.91 | 1.74 | 1.79 |
| 19 | 3 | 3.62 | 4.05 | 1.93 | 2.12 |
| 20 | 3 | 3.40 | 3.39 | 1.77 | 1.74 |
| 21 | 5 | 3.83 | 4.33 | 1.66 | 1.89 |
| 22 | 6 | 3.47 | 3.68 | 1.39 | 1.72 |
| 23 | 6 | 3.99 | 4.35 | 1.86 | 2.11 |
| 24 | 6 | 2.60 | 2.99 | 1.27 | 1.49 |
| 25 | 6 | 2.26 | 2.56 | 1.06 | 1.14 |
| 26 | 6 | 2.33 | 2.53 | 0.95 | 1.16 |
| 27 | 6 | 1.96 | 2.32 | 0.97 | 1.19 |
| 28 | 7 | 3.55 | 3.64 | 1.55 | 1.59 |
| 29 | 7 | 2.75 | 2.82 | 1.27 | 1.35 |
| 30 | 7 | 3.03 | 2.85 | 1.56 | 1.57 |
| 31 | 18 | 2.91 | 3.17 | 1.73 | 1.96 |
| 32 | 18 | 2.23 | 2.34 | 1.11 | 1.20 |
| 33 | 18 | 3.53 | 3.86 | 2.11 | 2.29 |
| 34 | 24 | 6.47 | 5.75 | 2.30 | 2.36 |
| 35 | 24 | 5.74 | 5.12 | 1.91 | 1.77 |
| 36 | 24 | 8.26 | 9.01 | 3.07 | 3.08 |
| 37 | 24 | 11.66 | 12.16 | 3.73 | 4.26 |
| 38 | 26 | 4.66 | 4.44 | 1.98 | 1.95 |

Table S3: SUV Repeatability metrics for 38 lesions of the *n* = 9 patients in cohort-1.

| Metric | SULmax | SUVpeak |
| --- | --- | --- |
| Min, max (g/mL) | 1.4, 8.8 | 1.3, 10.2 |
| $\bar{\boldsymbol{d}}$ ± Standard Deviation, $\mathbf{s}_{\boldsymbol{\Delta}}$ | 0.15 ± 0.27 | 0.20 ± 0.33 |
| *d_i_* min, max (g/mL) | -0.61, 0.56 | -0.42, 1.22 |
| Mean % difference, $\boldsymbol{\%}\bar{\boldsymbol{\Delta}}$ [-CI, +CI] (%) | 4.8 [1.0, 8.7] | 5.9 [0.5, 11.3] |
| Metrics with Bias Removed |  |  |
| Asymmetric Lower RC, *-*RC [-CI, +CI] (%) | -14.7 [-17.6, -11.6] | -14.0 [-18.7, -9.0] |
| Asymmetric Upper RC, *+*RC [CI, _CI] (%) | 17.2 [13.2, 21.4] | 16.3 [10.0, 23.1] |
| ${\boldsymbol{w}\mathbf{CV}}_{\boldsymbol{\Delta}}$ [-CI, +CI] (%) | 5.9 [4.6, 7.2] | 5.6 [3.5, 7.8] |
| Metrics with Bias Included as Variability |  |  |
| Asymmetric Lower RC including bias (*-*RC*_0_*) | -16.6 [-20.3, -12.9] | -17.0 [-24.9, -8.4] |
| Asymmetric Upper RC including bias (*+*RC*_0_*) | 20.0 [14.7, 25.4] | 20.6 [9.2, 33.1] |
| Within Subject COV including bias (*w*CV*_∆0_*) | 6.8 [5.1, 8.5] | 7.0 [3.2, 10.9] |

The parameters used in the calculation of repeatability under both bias assumptions appears in Table 1 for SUVmax and SULpeak, and in the table above for SULmax and SUVpeak using the log-transformed measures. The tables present a comparison of estimates of repeatability parameters with and without bias included as part of the variability based on 38 tumors in 9 patients for the various measures of SUV using log-transformed measurements.

Table S4: Cohort-2 patient and scan characteristics.

|  |  | Breast | Bone |  |  | FDG Scan Differences | | |
| --- | --- | --- | --- | --- | --- | --- | --- | --- |
| Case |  | Cancer | Cancer | ER/PR/Her2 | Treatment | ∆Days | ∆Uptake | ∆Dose |
| ID | Age | Type | Phenotype | Status | Therapy | (days) | (min) | (%) |
| 1 | 49 | Ductal | lytic | +/+/- | Endo | 183 | 0.6 | 0.2% |
| 2 | 65 | Ductal | sclerotic | +/+/- | Chemo | 81 | 5.7 | 9.6% |
| 5 | 41 | Ductal | lytic | +/+/- | Chemo | 190 | 0.8 | 14.6% |
| 6 | 50 | Ductal | unknown | +/+/+ | Chemo/Endo | 175 | 0.4 | 18.1% |
| 7 | 44 | Ductal | sclerotic | -/-/+ | Chemo/Endo | 200 | 9.4 | 4.8% |
| 8 | 44 | Ductal | lytic | +/+/- | Endo | 133 | 4.6 | 1.4% |
| 10 | 60 | Ductal | sclerotic | -/-/+ | Chemo/Endo | 84 | 0.2 | 4.3% |
| 11 | 34 | Ductal | lytic | +/+/+ | Endo | 217 | 4.9 | 15.1% |
| 12 | 76 | Ductal | unknown | +/+/- | Endo | 104 | 0.9 | 5.9% |
| 14 | 55 | Ductal | mixed | +/+/- | Endo | 203 | 4.3 | 7.0% |
| 19 | 37 | Ductal | mixed | +/+/- | Endo | 48 | 0.1 | 23.9% |
| 22 | 51 | Ductal | sclerotic | +/+/- | Chemo | 133 | 20.6 | 9.1% |
| 24 | 59 | Ductal | lytic | +/-/- | Chemo | 83 | 9.8 | 10.6% |
| 25 | 55 | Ductal | mixed | +/+/+ | Endo | 78 | 16.2 | 4.8% |
| 26 | 78 | Lobular | lytic | +/+/- | Endo | 175 | 14.9 | 0.5% |
| 27 | 47 | Ductal | sclerotic | +/+/- | Chemo | 126 | 25.0 | 13.9% |
| 30 | 47 | Ductal | mixed | +/+/- | Endo | 106 | 8.0 | 8.4% |
| 32 | 65 | Ductal | mixed | +/+/- | Endo | 84 | 3.6 | 6.8% |
| 33 | 62 | Ductal | mixed | +/+/+ | Endo | 142 | 10.8 | 20.9% |
| 37 | 58 | Lobular | sclerotic | +/+/- | Chemo | 152 | 1.3 | 2.8% |
| 38 | 75 | Ductal | lytic | +/+/- | Endo | 129 | 9.3 | 6.5% |
| 39 | 42 | Ductal | lytic | +/+/- | Endo | 122 | 0.1 | 6.2% |
| 40 | 64 | Lobular | lytic | +/-/- | Chemo | 119 | 14.2 | 24.7% |
| 41 | 72 | Lobular | sclerotic | +/+/- | Chemo | 774 | 45.0 | 1.0% |
| 42 | 59 | Mixed | lytic | +/+/u | Endo | 202 | 9.7 | 2.8% |
| 44 | 59 | Ductal | mixed | +/-/- | Endo | 89 | 2.4 | 18.9% |
| 45 | 44 | Ductal | mixed | -/-/- | Chemo | 146 | 0.4 | 0.4% |
| 50 | 91 | Ductal | lytic | u/u/u | Endo | 123 | 3.6 | 14.7% |

Case IDs 25-50 were scanned on the same PET/CT Scanner. *Estrogen receptor (ER), progesterone receptor (PR) and human epidermal growth factor receptor 2 (Her2) status is either present (+), absent (-) or unknown (u). ^†^Therapy was classified as either endocrine (Endo) or chemotherapy (Chemo). ^‡^FDG scan differences are ∆Days is the number of days between baseline and post therapy scans, ∆UT is the difference in the uptake time between injection and scanning for the 2 scans, and ∆Dose is the difference in injected dose between the scans

Table S5: Target lesion response characteristics for the 28 patients in Cohort-2.

|  |  |  | Meet | Meet |  |  |  |
| --- | --- | --- | --- | --- | --- | --- | --- |
|  |  |  | PERCIST | mPERCIST | New |  | Disease |
| Case | %∆SUVmax | %∆SULpeak | threshold? | threshold? | Lesions? | Evaluable? | Status |
| 1 | -13% | -12% | Yes | Yes | No | Yes | SD |
| 2 | -41% | 54% | No | No | No | No |  |
| 5 | -14% | 9% | Yes | Yes | Yes | Yes | PD |
| 6 | 14% | 15% | Yes | Yes | No | Yes | SD |
| 7 | -57% | -53% | No | No | No | No |  |
| 8 | -83% | -83% | Yes | Yes | No | Yes | PR |
| 10 | -31% | -40% | No | Yes | No | Yes | PR |
| 11 | -19% | -5% | Yes | Yes | No | Yes | SD |
| 12 | -42% | -34% | No | Yes | No | Yes | SD |
| 14 | -12% | 123% | No | Yes | Yes | Yes | PD |
| 19 | -62% | -58% | Yes | Yes | No | Yes | PR |
| 22 | -22% | 55% | Yes | Yes | No | Yes | PD |
| 24 | -3% | 133% | Yes | Yes | No | Yes | PD |
| 25 | -67% | -56% | Yes | Yes | No | Yes | PR |
| 26 | -83% | -80% | Yes | Yes | No | Yes | CR |
| 27 | -3% | 12% | Yes | Yes | Yes | Yes | PD |
| 30 | -56% | -63% | Yes | Yes | Yes | Yes | PD |
| 32 | -37% | 56% | Yes | Yes | No | Yes | PD |
| 33 | -29% | -9% | Yes | Yes | No | Yes | SD |
| 37 | -22% | -8% | Yes | Yes | No | Yes | SD |
| 38 | -78% | -79% | Yes | Yes | No | Yes | CR |
| 39 | -49% | 66% | Yes | Yes | Yes | Yes | PD |
| 40 | 30% | 7% | Yes | Yes | Yes | Yes | PD |
| 41 | -52% | 21% | Yes | Yes | Yes | Yes | PD |
| 42 | -17% | 104% | Yes | Yes | No | Yes | PD |
| 44 | 22% | 24% | Yes | Yes | Yes | Yes | PD |
| 45 | -61% | -58% | Yes | Yes | No | Yes | PR |
| 50 | -22% | -25% | No | Yes | No | Yes | SD |

Table S5 shows the response following therapy of the SUVmax and SULpeak values of the index lesion for each patient, the PERCIST/mPERCIST threshold status, the presence of new lesions, and evaluability of disease status. Of the two patients that were not evaluable, one had no lesions in either scan and the other had no lesion activity greater that the PERCIST or mPERCIST threshold. Disease status is complete response (CR), stable disease (SD), partial response (PR) and progressive disease (PD). PD is determined by the change in PET parameters greater than the upper (URC) and response (PR, CR) is less than the lower (LRC) repeatability coefficients. PD can also be determined by the presence of new lesions in the post therapy FDG scan, regardless of the change in SUV, PERCIST/mPERCIST threshold criteria or PERCIST inclusion criteria.

Figure S1.

Bland-Altman plots for cohort-1 patients

Figure S1: Bland-Altman plots of the average tumor values for the 9 individual patients of cohort-1. The size of each patient marker represents the number of lesions averaged for that patient, which ranges from 1 to 9 lesions. Top: SUVmax. Bottom: SULpeak. Left: Test-retest difference versus average value. Right: Differences of the natural logarithms. Dashed lines are the mean difference and the upper and lower limits of agreement (LOA_0_).

Figure S2.

Normality plot of test-retest tumors

Figure S2. Normal Q-Q Plot of log-transformed SULpeak values for 38 tumors from the test-retest cohort 1 showing normality. A two sample Kolmogorov-Smirnov test showed the distribution to be consistent with a normal distribution (p = 0.88). Similar results were obtained for log-transformed SUVmax values.

References

1. Velasquez, L.M., R. Boellaard, G. Kollia, et al. Repeatability of 18F-FDG PET in a multicenter phase I study of patients with advanced gastrointestinal malignancies. *J Nucl Med.* 50(10): 1646-54. 2009.

2. Weber, W.A., C.A. Gatsonis, P.D. Mozley, et al. Repeatability of 18F-FDG PET/CT in Advanced Non-Small Cell Lung Cancer: Prospective Assessment in 2 Multicenter Trials. *J Nucl Med.* 56(8): 1137-43. 2015.

3. Lodge, M.A. Repeatability of SUV in Oncologic (18)F-FDG PET. *J Nucl Med.* 58(4): 523-532. 2017.

4. Lipsitz, S.R., N.M. Laird, and D.P. Harrington. Using the jackknife to estimate the variance of regression estimators from repeated measures studies. *Communications in Statistics - Theory and Methods.* 19(3): 821-845. 1990.
